# The Implementation of Narrative Exposure Therapy (NET) for Transgender and Gender Diverse Adolescents and Young Adults

**DOI:** 10.1007/s40653-023-00530-4

**Published:** 2023-03-13

**Authors:** Jamie M. Julian, Jordan I. Held, Karen Hixson, Bridgid M. Conn

**Affiliations:** 1 Division of Adolescent and Young Adult Medicine, Children’s Hospital Los Angeles, Los Angeles, CA USA; 2 Portland, OR Portland, USA; 3 Division of Pediatrics, Keck School of Medicine, University of Southern California, Los Angeles, CA USA

**Keywords:** Trauma, Post-Traumatic Stress Disorder, Adolescents, Treatment, Transgender

## Abstract

**Purpose:**

There is limited information available regarding the use of trauma modalities within the transgender and gender diverse community (TGD) to address gender-based trauma, including discrimination and invalidation, particularly for adolescents and young adults (AYA). The purpose of this paper is to describe a novel treatment approach to addressing post-traumatic stress disorder (PTSD) symptoms within TGD AYA, inclusive of gender-based trauma.

**Methods:**

Narrative Exposure Therapy (NET) was implemented as a brief intervention for TGD AYA who had a positive screening for PTSD symptomatology. Measures were used to assess PTSD symptoms, as well as changes in self-perceived resilience and positive well-being. Two case vignettes are provided to demonstrate the adaptations made to be responsive to the unique needs of TGD AYA for trauma processing.

**Results:**

Preliminary outcomes from two case studies indicate the strength of NET when working with TGD AYA who face multiple traumatic events and continue to experience invalidation.

**Conclusion:**

NET shows promise as an effective brief intervention to reduce PTSD symptomology and increase resiliency in TGD AYA.

## Introduction

Transgender and gender diverse (TGD) individuals are those who do not identify with their sex designated-at-birth. These umbrella terms can be intended to encompass those who identify as a different gender identity outside of cisgender (i.e., individuals whose gender identity is congruent with their sex designated-at-birth), including individuals who do not identify within western concepts of a transgender identity (Adams et al., 2017). Within this article, the authors utilize these terms to reflect the experiences of individuals of a diverse, heterogeneous population. There is a growing body of literature highlighting that the mental health of TGD adolescents and young adults (AYA) is not an aspect of their identity, but resulting from chronic negative, invalidating, and unsupportive experiences in their environment. Research has shown that TGD AYA have similar mental health outcomes as their cisgender siblings when living in an environment that is affirming (e.g., supportive, validating) of their gender identity (Olson et al., 2016). Still, there exists a disproportionate representation of mental health conditions among TGD AYA, including depression, suicidality, self-injurious behavior, and suicide attempts (Connolly et al., 2016). One study of post-traumatic stress disorder (PTSD) among transgender youth showed that 50% experienced depression (compared to 20% of cisgender youth); that 26.7% were diagnosed with anxiety disorder (vs. 10% of cisgender youth); and that transgender youth were almost 3 times more likely to have attempted suicide compared to their cisgender peers (Reisner et al., 2015).

### Socio-Ecological Models of Stress and Trauma Among TGD AYA

A significant percentage of TGD AYA experience multiple and chronic traumatic events (Ryan & Rivers, 2003). Some of this trauma is overt, including verbal, physical, and sexual assaults. Some of this trauma is covert and more insidious, including lack of acceptance by family, friends, peers, and their community as their authentic selves. The ongoing epidemic of violence against the transgender community, rooted in transphobia, has been identified as a prolific and burgeoning public health crisis in the United States (James et al., 2016). In 2020, a survey conducted by the Trevor Project found that 40% of TGD AYA reported that they had been harmed or physically threatened because of their gender identity (Trevor Project, 2020), which aligns with previous research that has shown similar outcomes of physical violence among TGD adults (Kenagy & Bostwick, 2005; Testa et al., 2012). Multiple studies have shown the correlation of physical violence and peer bullying and rates of PTSD symptomology among AYA (D’Augelli et al., 2005; Herek & Garnets, 2007; Mustanski et al., 2010). This overt and covert trauma during formative developmental stages highlights the need to understand and address the chronic attack on one’s sense of safety for TGD AYA (Courtois, 2014). For many TGD AYA, these traumatic experiences are ongoing.

**Fig. 1:**
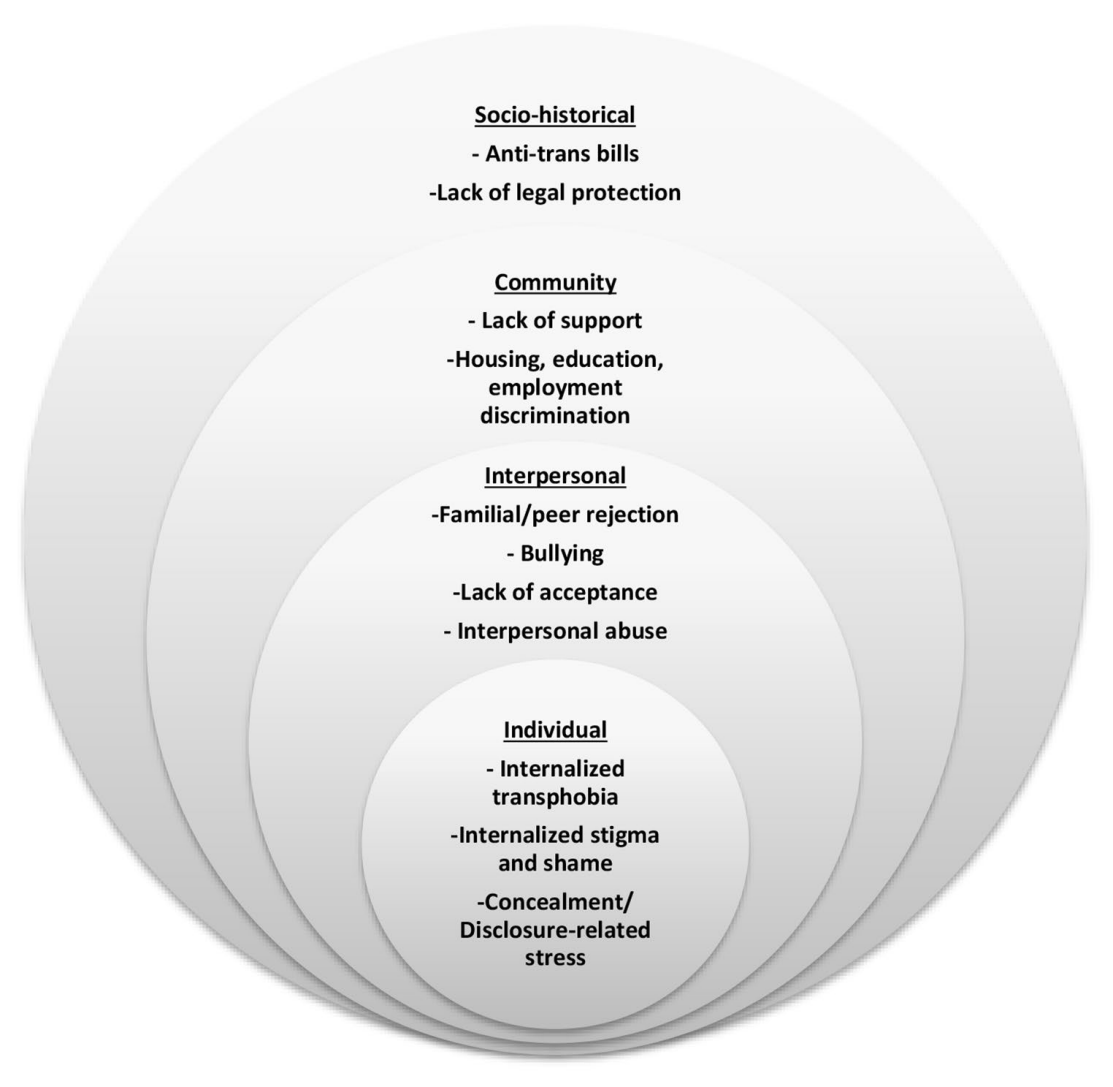
Socio-ecological model of gender minority stress for youth

The adapted minority stress model highlights the unique contributions of stigma, prejudice, and discrimination on the mental health of gender minority individuals from a socio-ecological framework (Fig. 1). The model is comprised of multiple levels of stress associated with one’s minority status including proximal and distal factors, reflecting internal stressors, such as identity concealment, internalized stigma, and beliefs of rejection, and external stressors, such acts of discrimination, systemic oppression, and violence, respectively (Hendricks & Testa, 2012). While the impact of physical threat and lack of safety are no doubt traumatic, the effects of prejudice, discrimination, and intolerant environments can create extreme fear by attacking one’s sense of safety, belief systems, and identity (Richmond et al., 2012). A growing literature has aimed to identify and describe the mental health challenges and needs of TGD individuals with the majority of studies providing supportive evidence of the minority stress model (Brennan et al., 2017). More specifically, findings have shown that TGD individuals experience high rates of psychological stress (i.e., anxiety, depression, somatization, suicidal ideation) associated with experiencing stigma, discrimination, and systemic oppression, particularly for individuals with intersecting identities that are marginalized, such as ethnic/racial, gender, and sexual minority statuses (e.g., Bockting, et al., 2013; Valente et al., 2020). TGD individuals experience layers of stress and trauma from bullying, lack of environmental support in school, work and community, as well as policies and legislation that seek to exclude individuals in the community from being able to use public spaces, engage in extracurricular activities, or receive protection from school and workplace harassment and discrimination (Rood et al., 2016). The impact of systemic oppression, gender-based violence, anti-trans legislation efforts, criminalization of healthcare, attack on Title IX protections, and limited providers with knowledge and competence to provide affirming care are all factors posited to contribute to the health inequities experienced by TGD individuals (Lefevor et al., 2019). These stressors have only increased in 2021 with a record number of proposed and enacted legislation targeting the rights and welfare of TGD individuals (ACLU, 2021).

Historically, therapy for TGD individuals has been rooted in the idea of diagnosis and pathology, communicating stigma and shame associated with societal perceptions of TGD identity as a mental illness with a need for treatment (Ault & Brzuzy, 2009; Lev, 2006). Beliefs regarding provider bias have been identified as a factor influencing the implementation of interventions within the TGD community. A recent report found that 46% of TGD AYA believed they could not find a competent mental health provider (Trevor Project, 2020). However, due to the fact that TGD AYA, as well as adults, are sometimes still required to obtain letters of support for medically necessary care, the relationship with mental health providers is often unbalanced and lacking safety (Mizock & Lewis, 2008). Thus, if an individual fears that self-disclosure regarding mental health might jeopardize their ability to receive a letter of support and access services, they may choose not to disclose, leading to further delay in treatment of mental health needs. These experiences, along with a higher prevalence of mental health issues that are endemic among TGD AYA, indicate an imperative need to develop gender-affirming approaches to mental health treatment. While there are increasing interventions developed to meet the needs of LGBTQ + youth (e.g., Project Youth AFFIRM; Craig & Austin, 2016) and transgender individuals (e.g., Trans-Affirmative Cognitive Behavioral Therapy; Austin & Craig, 2015), there remains a paucity of studies on the development and evaluation of evidence-based trauma treatments for TGD AYA.

## Narrative Exposure Therapy

Narrative Exposure Therapy (NET) is a trauma-focused, evidence-based intervention that utilizes narration, exposure, and processing to directly address the thoughts, feelings, and memories associated with each traumatic event (Schauer et al., 2011). The use of narration or storytelling is not a culturally-bound concept and its universal approach can be used with those unfamiliar or mistrustful of western approaches to therapy (Schauer et al., 2011). Narration has also been identified as one of the most effective pieces of a trauma intervention by clinicians and consumers in a qualitative study of Trauma-Focused Cognitive Behavioral Therapy (TF-CBT; Dittmann & Jensen, 2014). NET was developed to be similar to exposure-based and testimonial therapies and embodies key CBT principles, such as guided discovery and open-ended questioning to support identifying automatic thoughts and underlying core beliefs tied to the trauma(s). NET was designed as a manualized brief treatment intervention for individuals who have experienced multiple traumas and are still living in unstable environments. Unlike other trauma-focused interventions that ask participants to rank or select the index event, NET offers the opportunity to address multiple events that could be uniquely different (Schauer et al., 2011).

NET initially provides psychoeducation on PTSD and a groundwork for participants to understand the rationale behind the intervention. Participants are then asked to create a lifeline and identify traumatic memories as “stones” on their lifeline and moments of resiliency as “flowers”. In future sessions, each stone is processed, such that the narrative is reread to the participant at the beginning of every session to continue the exposure and habituation process and identify gaps in information or details. After all stones have been processed and the participant has been able to reconsolidate the memories, the clinician and participant close the intervention with “future flowers”, which is an exercise focused on supporting the client to reflect on their NET process, explore hopes for the future, and consider new perceptions of their self and their experiences within the context of healing. There are two primary goals of NET: (1) decrease PTSD symptomology through exposure and (2) create a cohesive chronological narrative utilizing contextual details, sensory, emotional, cognitive, and physiological information (Schauer et al., 2011). NET has been shown to be effective in several controlled trials at reducing PTSD and other trauma-related symptoms (Bichescu et al., 2007; Robjant & Fazel, 2010). It is also shown to have efficacy in working with chronically traumatized youth (Fazel et al., 2020; Ruf et al., 2010; Schauer et al., 2017). NET was initially designed to work with refugee populations and has been shown to have efficacy with many ethnic/racial groups. NET has been used in multiple randomized controlled studies with ethnically diverse populations and was found to be effective in PTSD symptom reduction (Jongedijk, 2014). A recent meta-analysis of NET found significant and sustained positive outcomes for refugees and trauma survivors (Lely et al., 2019; Lange, 2020) described a pilot of NET with transgender veterans, including considerations for providing gender-affirming mental health treatment and efficacy in utilizing NET in processing trauma related to gender minority stress. Several studies have compared NET with other trauma-based treatments (Ruf et al., 2010; Schaal et al., 2009). For instance, a study examining the effectiveness of NET compared to Interpersonal Psychotherapy (IPT) found that NET was effective in reducing PTSD symptomatology and that symptoms had further resolved at 6-month follow-up unlike the IPT cohort (Schaal et al., 2009).

### NET as a Social Justice-Oriented Trauma Treatment

Several unique aspects of NET contribute to its utility as a social justice-oriented intervention for TGD AYA with PTSD. More specifically, the NET protocol includes both a therapeutic focus on treatment of an individual’s PTSD, as well as a sociopolitical focus on the aspects of oppression or political context that have contributed to the client’s trauma (Bichescu et al., 2007). The client-centered tenets of NET allow TGD AYA the opportunity to share their own story, work through and dispel gender myths often imposed upon them, and critique traditional forms of gender identity; all separate from the narrative their family and society often imposes upon them. NET supports the client to express their trauma narrative in their own words, which allows them to define the way they use their name, pronouns, and other descriptors to reference a younger version of themselves.

NET is designed to be a low barrier intervention for clinicians and participants due its low drop-out rates (Robjant & Fazel, 2010) and low implementation costs. Unlike many other trauma modalities, which require a lengthy and costly training and certification processes, NET training can be completed in two days or less and at a much lower cost, typically less than $3,000 for an entire agency. Average costs were calculated for seven commonly implemented evidence-based treatments, including three trauma treatments: Prolonged Exposure, Cognitive Processing Therapy, and Trauma-Focused Cognitive Behavior Therapy. The authors found the total costs associated with these three modalities ranged from $2,231.32 to $7,418.61, with PE being the most expensive and TF-CBT being the least and closest in cost to NET (Okamura et al., 2018). In addition, there are no training certification bodies for NET and no ongoing consultation requirements. This has major implications for low-resourced community mental health clinics that may not be able to afford costly training initiatives, especially with higher turnover rates among staff (Brabson et al., 2020). NET’s fundamental principles support individual autonomy and personal strengths over adverse events; a key goal in supporting TGD AYA. These guiding principles posit promising outcomes for the reclamation of identity pride and reduction in trauma responses for TGD AYA (Gwozdziewycz & Mehl-Madrona, 2013).

## Aims

In the current article, we present two case studies of NET with TGD AYA in a community mental health clinic. We describe our considerations and modifications of a culturally responsive, gender-affirming brief treatment to reduce PTSD symptomatology among a community that experiences greater rates of trauma and minority stress, as well as significant barriers to accessing treatment focused on addressing trauma experiences related to gender-based stigma, interpersonal rejection, and discrimination. To accurately assess for reduction in PTSD symptoms, we also describe adaptations to a frequently utilized measure of PTSD symptoms in youth to capture the broad range of trauma experiences for TGD AYA. The case studies describe the implementation of NET for two TGD AYA with complex trauma from economically disadvantaged backgrounds. The authors then describe unique aspects of the narrative development and exposure process for gender-based minority stress and trauma over the lifespan.

## Methods

### Participants

The clinical care described in these case studies was conducted within a gender health clinic embedded within a large tertiary care pediatric medical setting in the Southwestern United States. TGD AYA present to the clinic to access medical and mental health care, as well as other resources. The purpose of the Trans Community Trauma Treatment Center for Children and Adolescents (TCTTC), which is located within the gender health clinic, is to provide a critical opportunity to expand and refine best practices for a population that has a high rate of exposure to complex trauma, many of whom are also disproportionately unable to access mental health services due to being uninsured or under-insured or of lower income status. Improving our ability to appropriately identify, treat, and mitigate the complex trauma experienced by TGD AYA will have vast implications for overall health and quality of life. Clients are TGD AYA, ages 10–21, who participate in a no-cost brief therapy intervention. To receive NET, clients from the gender health program are screened for PTSD symptoms at intake (described further below) or may be referred by providers for patients that are already receiving medical care. Clients described in these case vignettes provided a separate consent to have their data presented as part of this case series.

### Measures

#### Assessment of Trauma Symptoms

The Primary Care PTSD (PC-PTSD-5) is a five-item screener used to screen for PTSD symptoms based on DSM-5 criteria in primary care settings (Prins et al., 2015). The screener is designed to capture exposure to traumatic events and associated symptomatology. During clinic intakes, all clients are asked to respond, based on the last month, if they have experienced any of the following symptoms: nightmares, intrusive thoughts, hypervigilance, dissociation, and guilt/blame. A cut-off score of three or more was used to establish preliminary eligibility for the intervention (Prins et al., 2015), which was then followed by an additional assessment using the UCLA Post-Traumatic Stress Disorder Reaction Index for DSM-5.

**Fig. 2:**
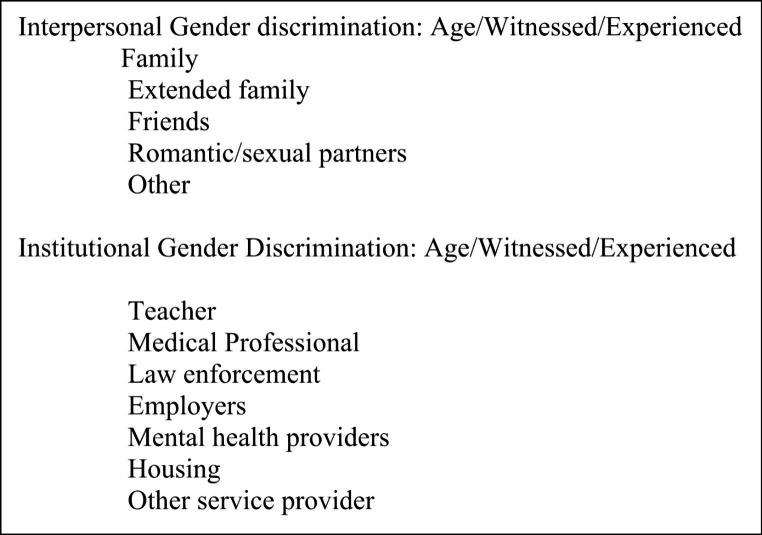
Additional questions added to the UCLA PTSD Reaction Index

The UCLA Post-Traumatic Stress Disorder Reaction Index for DSM-5 (PTSD-RI-5) is a widely utilized and validated measure for screening and assessing children and adolescents for symptoms of post-traumatic stress associated with a range of potentially stressful and traumatic life experiences (Doric et al., 2019). The criteria for PTSD were revised in 2013 in the Diagnostic and Statistical Manual Fifth Edition (DSM-5; American Psychiatric Association, 2013), which include: exposure to a potentially traumatic event (directly or vicariously through witnessing or learning about the event), intrusive symptoms (e.g., flashbacks, nightmares), avoidance of cues (e.g., people, places, or things that remind one of the event), negative alterations in cognitions and mood (e.g., dissociation, distorted cognitions related to self-blame and negative beliefs about one’s self, others, and the world), and arousal and reactivity (e.g., hypervigilance, recklessness). The recently updated DSM-5-TR maintains these additional changes to the PTSD criteria established in 2013 and allows for the inclusion witnessing of trauma to include indirect sources like “electronic media, television, movies, or pictures” for youth 6 years and younger (DSM-5-TR; American Psychiatric Association, 2022). While this tool appears to have solid validity and reliability (Doric et al., 2019), to date, there have been no similar explorations of the clinical utility for this tool with TGD AYA. In our clinic protocol, we provide recommended modifications for the trauma history profile within the UCLA PTSD-RI-5, considering the broader conceptualization of potentially traumatic experiences for TGD AYA. The modified PTSD measure contains two additional items reflecting gender-based traumatic experiences, including bullying, discrimination, and abuse at the interpersonal and institutional levels (Fig. 2).

#### Assessment of Resilience, Coping, and Positive Well-Being

The Connor-Davis Resilience Scale (CD-RISC-25) is a 25-item measure designed to capture and assess psychological resilience (Davidson, 2018). It was developed to identify the likelihood of individuals’ ability to manage and cope with stressful situations, traumatic events, and loss, acknowledging that psychological resiliency can fluctuate over time. Clients were asked to answer statements based on the last month (e.g., “I am able to adapt to change”). Scoring is based on a five-point scale (e.g., 0 = not at all true, 4 = true nearly all of the time). The possible total scores range from 0 to 100 with higher scores being an indicator of high psychological resilience (Davidson, 2018).

The Flourishing Scale is an 8-item tool used to measure positive functioning, including self-esteem, relationships, purpose, and optimism (Diener et al., 2009). This measure was included to establish the psychological well-being of participants from their perspective. Scoring is based on a 7-point Likert scale (i.e., 1 = strongly disagree, 7 = strongly agree), with a range of 8–56. Higher scores indicate higher likelihood of psychological wellbeing (Diener et al., 2009). A recent study of Canadian adolescents supported the use of the Flourishing Scale as a valid measure of psychological well-being (Romano et al., 2020).

#### NET Intake and Therapy Protocol

Clients participated in a clinical intake (in-person or via telehealth) to gather demographic information and complete the initial measures (described above). All therapy sessions were provided via a HIPAA-compliant telehealth platform (i.e., WebEx). Assessment measures were conducted at baseline, discharge, and at 6-months following discharge. While treatment is generally brief, clients had the option to extend based on the number of “stones” to be processed in treatment and other ongoing needs addressed during the course of treatment (e.g., suicidality, new traumatic events). For these case studies, clients had an average of 12 sessions. The protocol for treatment was developed with consideration for guidelines in conducting NET via telehealth (Kaltenbach et al., 2021).

### Case Series

#### Participant #1

A 22-year-old non-binary, Caucasian, designated-female-at-birth individual who had a positive screening for PTSD symptomatology on the PC-PTSD-5 was referred to NET. This client had publicly funded insurance and had been previously seen for mental health services through philanthropic funding due to a lengthy waitlist for providers that accept publicly funded insurance plans within the community. The client reported not being connected to any family and was receiving housing support from a different service provider. Due to limited community mental health providers who accept publicly funded insurances, lack of gender-affirmative mental health providers, and mistrust of cisgender-centered institutions, this client had never addressed their trauma, which had begun in early childhood. The client presented with hypervigilance, intrusive thoughts, nightmares, and somatic response when activated by perceived threats. The client was provided an overview of NET and reported that they wanted to try something different after being in traditional talk therapy for years and not feeling that they were making significant progress in reducing disruptive thoughts and feelings associated with their trauma. The client was connected to a licensed clinical social worker for the intervention and treatment was administered via telehealth within the context of the ongoing COVID-19 pandemic.

The initial stages of treatment included baseline assessment and creation of the lifeline. Based on the baseline assessment, the client did not meet the full criteria for a PTSD diagnosis based on the UCLA PTSD-RI; however, they did report symptoms of intrusion, negative cognitions and mood, and dissociative symptoms. The client’s lifeline included events prior to them coming out as well as after coming out as non-binary. Gender played a significant role in all of their identified stones from how they referenced their younger self to feeling targeted due to their behavior that did not align with expected gender norms. Gender-based discrimination was captured in the baseline trauma inventory, including interpersonal, as well as institutional, gender-based discrimination. The client had experienced multiple traumatic events in addition to gender-based discrimination, including interpersonal violence and was interviewed by the police as a minor and as an adult. They recalled these interviews as points of shame, including invalidation of their gender and perceived judgment that led to them not feeling comfortable sharing or seeking additional help. When actively engaged in treatment, NET allowed the client to retell their experience without judgment and scrutiny of details. Processing the abuse was activating for the client and they experienced several physical responses, including nausea, sweating, and other physical sensations. Upon completion of three sessions of processing, the client reported no physical sensations, had reached habituation, and was able to move to the next identified stone in their lifeline. In the final stages of treatment, the client engaged in the future flowers exercise and discharge measures.

In creating their “future flowers”, the client was able to identity newly found hope and optimism about their ability to engage in life (i.e., “I can do the things I want to do; I don’t need to always be afraid”). When asked what they learned about themselves while in NET they responded, “I am an important person, my feelings are valid”. The client completed 14 sessions in a 4-month period and upon discharge, no longer reported significant symptoms of intrusion, negative cognitions or mood, or dissociation. The client reported significant increases in self-perceived resiliency, coping, and positive well-being (see Table 1).

#### Participant #2

A 19-year-old, Caucasian, designated-male-at-birth, transgender female presented for treatment with a significant trauma history, as well as a history of anxiety, depression, and suicidality. The client stated that her trauma caused impairments in her activities of daily living and impacted her ability to foresee a future sense of self. She verbalized multiple adverse events throughout her childhood; however, the most clinically significant event pertained to abuse by her biological father and lack of appropriate caregiver. The client screened positive on the PC-PTSD-5 and was provided psychoeducation on trauma and the rationale behind NET. She agreed to participate and completed baseline measures. She was connected to a supervised clinical social worker to provide the intervention. During intake, the client described symptoms of intrusive ideation, as well as experiences of depersonalization, or feeling disconnected from herself. She met full criteria for PTSD based on the UCLA PTSD-RI.

Prior to participating in NET, the client stated she had never discussed her history of abuse as it was “too painful to share.” She verbalized that this event “holds me back” and makes her feel “like I can’t live,” even years later. In the initial stages of treatment, the client created her lifeline and identified the stones she felt were the most impactful. As treatment progressed, the client was very hesitant to start one of her trauma narratives. She stated that part of the pain involved the actual incident; however, the other aspect involved her perceived inability to discuss due to her gender dysphoria and how she relates to her sense of self, pre-medical transition. She recalled multiple aspects of the event as traumatic and scary; however, for her, one of the components of complexity involved her own feelings surrounding her genitals, which prior to NET, she was never able to acknowledge. She also identified never feeling comfortable talking to a mental health provider about these details due to her feeling like this would be used to dismiss her identity and prevent her from moving forward with her transition. During the narrative exposure process, the client verbalized the painful experience of being “trapped in the wrong body.” She recalled feeling a sense of hopelessness surrounding her body, her self-worth, and an overall feeling of “disgust and shame”, which was separate from her experience of abuse. Through the client’s narration of this painful trauma, she was able to verbalize, contextualize, and then separate the abuse from her experiences of gender dysphoria; something she was never able to do before recalling the event. The witnessing of this particular narrative was not only integral to her trauma processing, but also integral to her formation of a future sense of self. This process assisted her in separating her trauma from her dysphoria; thus, reducing self-blame while enhancing her sense of resilience. In the final stages of treatment, she was able to use this resilience to create her “future flowers” and reported having a different relationship with her transitioning body. Upon discharge, the client reported a reduction in her intrusive thoughts, as well as a reduction in feelings of depersonalization. The client completed the intervention in 10 sessions over a 3-month period. At discharge, she no longer met any of the criteria for PTSD based on the UCLA PTSD-RI and reported a significant increase in her resiliency and flourishing scores (see Table 1).

**Table 1:**
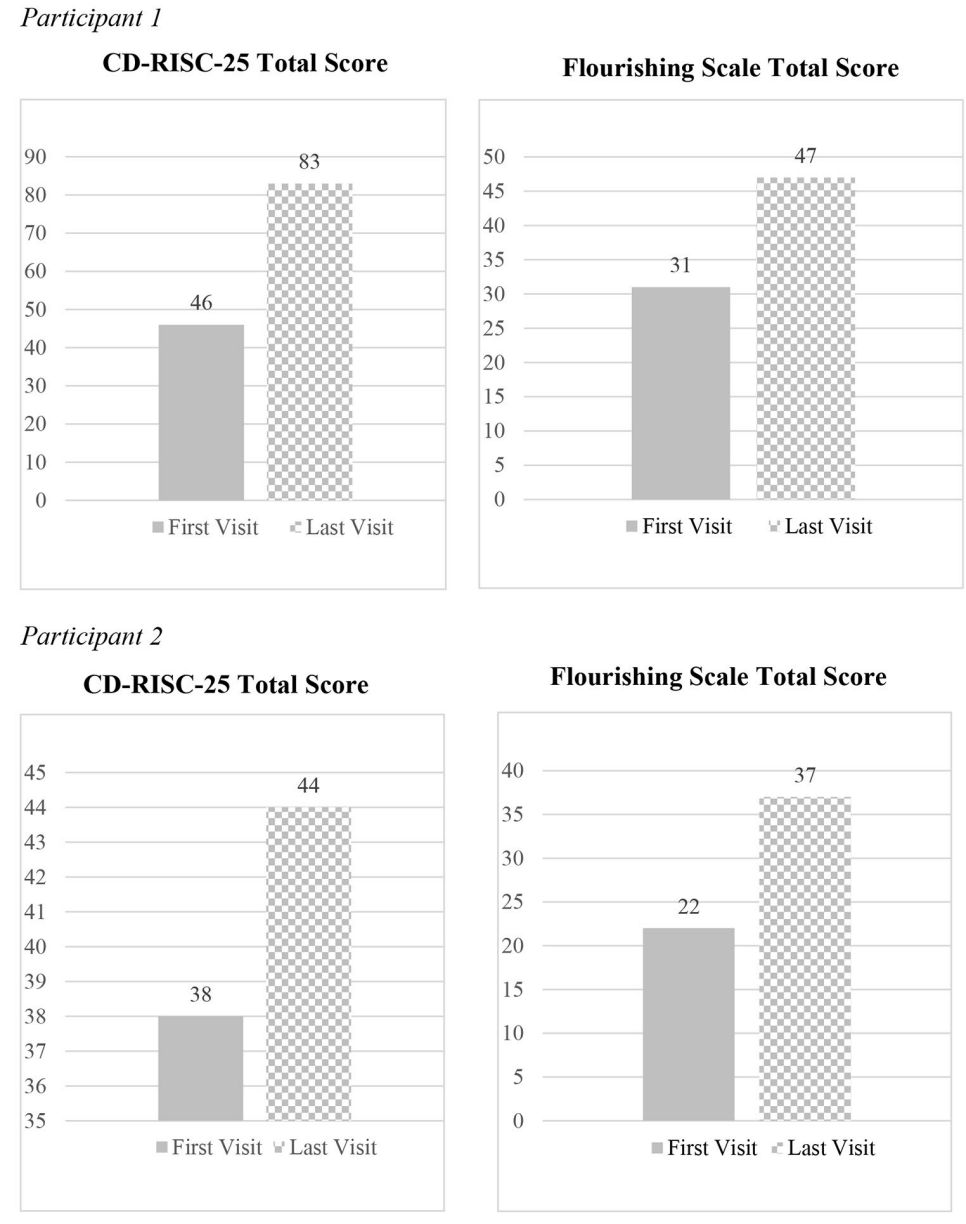
CD-RISC-25 and Flourishing Scale scores

## Discussion

The potential power of NET in working with TGD AYA is in the approach. Clients are the experts in their experience and are given the opportunity to reclaim their autonomy through their narratives, acknowledging the impact of these experiences on their development and finding resilience in their identities. Too often TGD AYA are faced with transphobia and invalidation from cis-centered providers who often do more harm though misguided intentions that inhibit a healing-centered journey. The research clearly indicates the importance of an intervention in addressing the unique needs of TGD AYA who face a myriad of barriers, oppression, and discrimination (Reisner et al., 2016). As research continues to try and fully understand the clinical presentation of insidious and complex trauma due to chronic invalidation, discrimination, and rejection, it is critical to identify ways to combat the barriers to appropriate care for TGD AYA. Preliminary findings from these case studies provide promising evidence of the ability of NET to reduce PTSD symptomatology through the reconstruction of memories and exposure-based processing with special considerations for gender-based trauma. NET’s low-barrier training for community mental health clinicians also has promise to provide brief, effective trauma modalities to those who historically have been unserved and underserved (Robjant & Fazel, 2010). The processes and outcomes of these case vignettes demonstrate the utility of a brief trauma treatment modality that can support a reduction in disruptive PTSD symptomatology. Further, these vignettes highlight the strength of NET in being adaptable to addressing multiple traumatic experiences among TGD AYA, which often are heightened by invalidation and questioning by non-affirmative service providers (e.g., police, clinicians).

### Future Directions

The benefits of NET continue to be identified as more studies aim to evaluate its effectiveness (Park et al., 2020). Like most inventions, NET is not without its limitations as a modality. NET places a heavy burden on the clinician by requiring clinicians to document the narrative as accurately as possible, capturing the very specific details of the traumatic experience. It is the authors’ experience that trauma-heavy work requires smaller caseloads and greater supervision in place to support secondary trauma processing. As NET is a brief treatment intervention that specifically targets trauma, it is not designed to directly address comorbid conditions that clients might be experiencing. Thus, some clients might require additional therapy after completion of NET. The majority of studies utilizing NET have had small sample sizes and have targeted specific populations making it difficult to generalize the results to other populations (Pabst et al., 2014). Therefore, it is critical to continue to evaluate the effectiveness of NET with different, diverse populations to fully understand the benefits and limitations of this intervention. The present article reflects the preliminary efforts to describe the adaptations of a brief trauma-focused intervention among TGD AYA, a historically marginalized and under-served community. Efforts to evaluate its implementation and effectiveness are under way.
